# Assisted neuroscience knowledge extraction via machine learning applied to neural reconstruction metadata on NeuroMorpho.Org

**DOI:** 10.1186/s40708-022-00174-4

**Published:** 2022-11-07

**Authors:** Kayvan Bijari, Yasmeen Zoubi, Giorgio A. Ascoli

**Affiliations:** 1 College of Science, Neuroscience Program, George Mason University, Fairfax, USA; 2 Center for Neural Informatics, Structures, & Plasticity, Krasnow Institute for Advanced Study, George Mason University, Fairfax, USA; 3 Bioengineering Department, Volgenau School of Engineering, George Mason University, Fairfax, USA

**Keywords:** Metadata management, Neuro-curation, Neuroinformatics, Natural language processing, Named entity recognition, Machine intelligence, Deep learning, Transformers

## Abstract

The amount of unstructured text produced daily in scholarly journals is enormous. Systematically identifying, sorting, and structuring information from such a volume of data is increasingly challenging for researchers even in delimited domains. Named entity recognition is a fundamental natural language processing tool that can be trained to annotate, structure, and extract information from scientific articles. Here, we harness state-of-the-art machine learning techniques and develop a smart neuroscience metadata suggestion system accessible by both humans through a user-friendly graphical interface and machines via Application Programming Interface. We demonstrate a practical application to the public repository of neural reconstructions, NeuroMorpho.Org, thus expanding the existing web-based metadata management system currently in use. Quantitative analysis indicates that the suggestion system reduces personnel labor by at least 50%. Moreover, our results show that larger training datasets with the same software architecture are unlikely to further improve performance without ad-hoc heuristics due to intrinsic ambiguities in neuroscience nomenclature. All components of this project are released open source for community enhancement and extensions to additional applications.

## Introduction

A named entity is anything that can be referred to with a proper name. Common named entities in neuroscience articles are animal species (e.g., mouse, drosophila, zebrafish), anatomical regions (e.g., neocortex, mushroom body, cerebellum), experimental conditions (e.g., control, tetrodotoxin treatment, Scn1a knockout), and cell types (e.g., pyramidal neuron, direction-sensitive mechanoreceptor, oligodendrocyte) [15]. The task of named entity recognition (NER) consists of identifying spans of text (*mentions*) that comprise named entities and tagging the entity [28]: for example, recognizing every time an article refers to tetrodotoxin treatment and tagging them as experimental condition. Entity recognition is a non-trivial task, due in part to the difficulty of segmentation, i.e., deciding what is or is not an entity and where its boundaries are in the text. For example, the term *Swiss* by itself is not a neuroscience-specific entity; however, *Swiss Albino* and *Swiss-Webster* are mouse strain names for common neuroscience animal models. Another challenge is the terminological vagueness of cross-type entities, as in *ganglion*, which is both a neuron type of the retina and an invertebrate brain region, and Golgi, which is both a cerebellar neuron and a histological stain.

NER plays a crucial role in information extraction and natural language processing (NLP). Nonetheless, most NER models and datasets are domain-specific (e.g., [12, 36]). These models are difficult to generalize, because the entity categorical needs differ across domains, and for many specific domains no suitable datasets are publicly available [6, 20].

A prominent approach for performing NER relies on pre-existing and domain-specific vocabularies to identify mentions of entities in the text. Dictionary-based models are widely used for their simplicity, as they can extract from a document all matched entities listed in a vocabulary. However, compiling accurate dictionaries requires expensive effort by domain experts, and comprehensive coverage of all relevant types of named entities often remains elusive [19]. Furthermore, vocabulary-based methods suffer from low recall because of their brittleness with respect to the multifarious variations of the target terms. The problem is especially serious in neuroscience due to the lack of standardized nomenclature and heterogeneity of abbreviation conventions in most relevant entity types, such an anatomical regions and experimental conditions, which make name recognition challenging even for human professionals [16]. In addition, the lack of community consensus on a common classification for key entities, particularly cell types, compounds the unsolved terminological problem with an even deeper conceptual ambiguity [40]. Nevertheless, dictionaries have been shown to enhance state-of-the-art NER systems by incorporating information about the similarity of the extracted entities with available terms [21, 34].

Deep learning has been applied to information extraction in neuroscience [39]. In general, deep learning approaches to NLP typically require mapping words from unstructured text into numerical vectors called word embeddings [31]. Word embeddings precomputed from large corpora may be beneficial in solving NER tasks [27]: instead of training a model from scratch, importing models pre-trained on related datasets (referred to as transfer learning) can lower the computational cost of training [44].

Bidirectional Encoder Representations from Transformers (BERT) is a word embedding model that employs bidirectional transformers for pre-training [11]. Transformers are deep neural networks that derive semantic and syntactic information from the contextual relation of each word with all other words in the sentence [42]. The transformers utilized by BERT process text bidirectionally from right-to-left and left-to-right at once. The pre-trained BERT can be fine-tuned to create competitive models for a wide range of downstream NLP tasks, including NER.

In this work, we pre-train the base BERT architecture on a large neuroscience corpus to increase its text mining efficacy in this domain. Then we fine-tune the resultant pre-trained language model (NeuroBERT) for the NER task of identifying and tagging mentions of neuroscience terms from peer-reviewed articles. Specifically, we augment our custom neuroscience information extraction algorithm with term statistics and curated ontologies from the open access repository of neural reconstructions, NeuroMorpho.Org [2]. We thus deploy a smart, context-aware, domain-specific knowledge suggestion engine that complements and interfaces with our previously developed literature [25] and metadata management systems [7] to foster the public availability of digital reconstructions of neural morphology.

## Materials and methods

The proposed system takes a sequence of paragraphs from a neuroscience article and extracts a list of metadata entities that best characterize the data described in the article. The core extraction mechanism is a sequence labeling algorithm, which requires training. Once the sequence labeling algorithm is trained, we take advantage of a probabilistic sentence classifier tuned with term statistics extracted from the publication and from NeuroMorpho.Org [4] to sort the extracted entities and provide a ranked metadata suggestion list. The following sections describe the algorithm, the training dataset, and explain the structure of the entity suggestion system.

### Corpus preparation and preprocessing

**Fig. 1:**
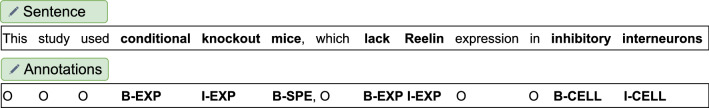
Sample sentence from a neuroscience article annotated with entities of interest in BIO format. B–X indicates the beginning of the new entity X in the word sequence, I–X presents the continuation of entity X in sequence, and O points to the words out of the area of interest

The data utilized in our metadata suggestion system consists of two main parts. The first part is the dataset prepared for training the sequence labeling algorithm. Starting from a corpus of over 2000 neuroscience articles processed for NeuroMorpho.Org, we selected 13,688 sentences via active learning [9]. We then used the open-source annotation software DataTurks (https://github.com/DataTurks) to manually annotate the sequence of words with nearly 40,000 target metadata labels of interest (Table 1). The length of the annotated output sequence (labels) is thus the same as that of the input sentence. For this NER annotation, we adopt the BIO format [35], a tagging scheme that captures both boundary and the named entity types (Fig. 1). All training data described above, including publication identifiers and annotated sentences, are available at https://gitlab.orc.gmu.edu/kbijari/neuroner-api/-/tree/master/data.

**Table 1:** List of neuroscience entities of interest for NeuroMorpho.Org with their abbreviated form that is used in this article along with an example of its type and their distribution in the annotated sentences

Entity	Abbreviation	Example	Count
Cell type	CEL	Interneuron	7549
Developmental stage	DEV	Adult	1243
Experimental condition	EXP	Control	9860
Sex or Gender	GEN	Female	685
Objective type	OBJ	Oil	156
Protocol	PRO	In vivo	1482
Reconstruction software	REC	Imaris	1209
Brain region	REG	Amygdala	8314
Slicing direction	SLI	Coronal	362
Species	SPE	Rat	4481
Staining method	STA	Biocytin	1402
Strain	STR	Wistar	3169
Total	–	–	39,876

**Fig. 2:**
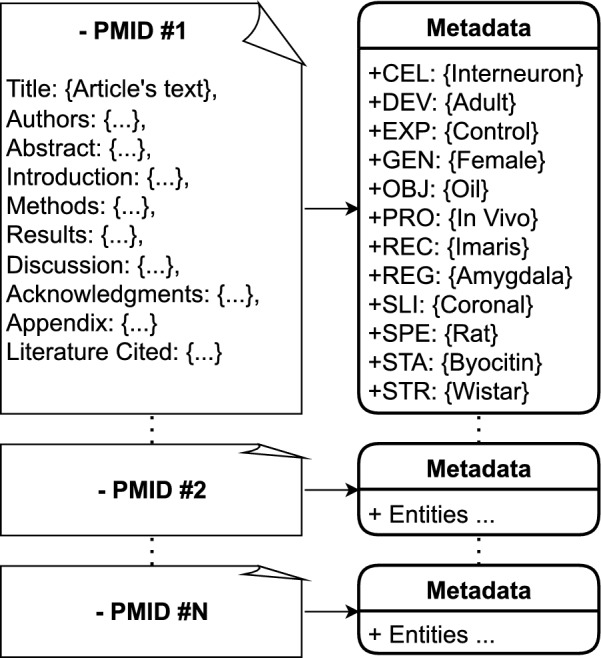
Schematic representation of the data for the metadata extraction task. Unstructured text of neuroscience articles are mapped to their corresponding metadata matching the NeuroMorpho.Org schema. Each article has a body of text extracted from PubMed or other publishers and is associated with a list of target metadata entities. The task of the algorithm is to find the target entities in the text of the article and create a list of suggestions ranked based on their relevancy

The second part of the data consists of metadata entities of specific collections of neural reconstructions publicly shared on NeuroMorpho.Org and associated with 812 published articles [7]. These highly curated metadata summaries constitute the gold standard for benchmarking the automated suggestion system (Fig. 2). It is important to note that typically not all NeuroMorpho.Org metadata are explicitly mentioned in the publication: certain entities (such as the name of the collection and the structural domains included in the data) are only provided directly by the dataset contributors at the time of submission to the repository [29]. Therefore, this work solely focuses on extracting the subset of metadata most commonly reported in publications (Table 1). These 12 metadata entities to be extracted can be logically grouped in three broad categories: animal, anatomy, and experiment, as briefly explained below.

The four metadata entities pertaining to the *animal* category specifies information about the subject of the study: the species, strain, sex, and developmental stage. This knowledge is the simplest to extract from the article, as the corresponding details are almost always clearly stated in the text of the publication.

The two metadata entities in the *anatomy* category represent the nervous system region and the cell type of the reconstructions described. These are the most difficult characteristics to recognize automatically for three main reasons. First, their semantics strongly depend on the species: many regions and cell types only exist in vertebrates (e.g., cerebellum and pyramidal cells) or invertebrates (e.g., antennal lobe and Kenyon cells). This provides tight contextual constraints that require considerable specialization for proper interpretation. The second reason is that anatomical entities can often be labeled according to different criteria, which typically vary based on the specific focus of the study. For example, regions can be partitioned functionally (e.g., visual vs. somatosensory vs. motor cortex) or structurally (e.g., occipital vs. parietal vs. frontal lobes); and cell types can be classified electrophysiologically (fast-spiking vs. regular spiking), or molecularly (calbindin-expressing vs. calretinin-expressing). These labels often overlap to a certain extent across criteria, considerably complicating the annotation task. The third challenge is that NeuroMorpho.Org divides both anatomical regions and cell types into three hierarchical levels, from generic to specific (e.g., hippocampus/CA1/pyramidal layer and interneuron/basket cell/horizontal). Not all hierarchical descriptors might be explicitly mentioned in the article, as authors often rely on the reader’s tacit knowledge for correct understanding. To overcome these obstacles, we take advantage of the substantial information contained in the manually curated metadata from thousands of publications by more than 900 labs as provided by NeuroMorpho.Org and its public metadata hierarchies [32]. Specifically, from these records we constructed a lookup table for individual terms listing correlations with other metadata dimensions, potential hierarchies, synonyms, and frequencies.

The last six metadata entities targeted by our suggestion system, belonging to the experiment category, describe methodological information: the preparation protocol, experimental condition, label or stain, slicing orientation, objective type, and the tracing software. These details are also relatively straightforward to extract, since peer-reviewed publications usually mention the experimental specifications explicitly.

If any metadata detail is not provided by the contributor nor mentioned in the publication, the corresponding entry is marked “Not reported” in NeuroMorpho.Org. Moreover, certain details are labeled as “Not applicable” depending on the specific preparation: for instance, if the experimental protocol is “cell culture”, then the slicing direction is not a relevant entity.

### Metadata extraction—problem definition & system architecture

Given a full text of a publication (P) captured from the publisher’s application programming interface (API), and an empty list of target metadata (M) dimensions (Table 1), the entity extraction task is to collect all metadata entities noted within the sentences of P and add them into the appropriate sub-list of M. Then, the algorithm must sort all sub-lists of M based on the relevance of the extracted entities.

**Fig. 3:**
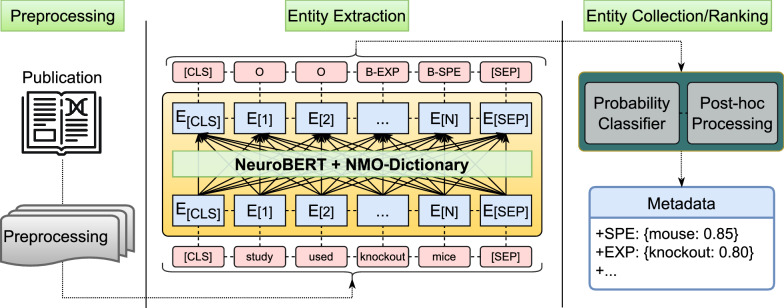
Overview of the architecture of the metadata extraction system used in this study. The full text of the publication is preprocessed, marked with sentence boundaries (CLS and SEP), and converted into sequences of words. Each sequence is then passed through the entity extraction algorithm to identify occurrences of metadata entities. The candidate terms are then post-processed and ranked based on relevance score

The metadata extraction architecture we designed to solve the above task consists of three main elements: preprocessing, entity extraction, and entity collection/ranking (Fig. 3).

Preprocessing starts by resolving all abbreviations mentioned in the full text using Hearst’s algorithm [37] and a list of common abbreviations collected throughout our practice on the NeuroMorpho.Org knowledge repository. Afterward, we replace all Latin numeric mentions with their corresponding Arabic numerals (e.g., i/ii → 1/2). Once this is done, we use the NLP toolkit [23] to break the text into paragraphs and sentences, next, sentences are tokenized into a sequence of words and punctuations. Having the full text of publication transformed into a list of word sequences, we can turn to tagging metadata entities.

The sequence-to-sequence algorithm to extract named entities consists of two sub-parts. First, NeuroBERT encapsulates the preprocessed sequence of words with special tokens ([CLS] and [SEP]) to demarcate the sentence boundaries and creates appropriate metadata tags for each term. Second, to ensure that all entities are fully extracted from the text, and nothing is missed, we cross-check the terms in the sequence for exact matches in the lookup table of NeuroMorpho.Org metadata terminology.

The above process produces labels for every term in all publication sentences. This dense annotation is then trimmed by removing labels that cannot be mapped to suitable target terms from the NeuroMorpho.Org lookup dictionary. Matching between extracted labels and NeuroMorpho.Org target term is assessed by Jaro similarity [18]. This metric measures edit distance between two strings in the [0, 1] range, with 1 indicating identical strings and 0 indicating no character in common between them. For our purpose, we set a similarity threshold of 0.85 to retain a label.

The result of this sequence-to-sequence processing is a list of candidate terms for each metadata entity type (category). At this stage, the terms within each entity type must be ranked to identify the most accurate metadata suggestion.

### Metadata entity ranking

To rank terms within the list of identified candidates for each metadata entity type, we assign to each candidate term a score that is a function of its occurrence frequency and location in the text, its usage rate in NeuroMorpho.Org, and the structure of the sentence in which it appears. Specifically, the score of each extracted entity is determined based on the following equation.

$$\begin{array}{c}Score\left(term,sec,sen\right)=\alpha \times Freq\left(term\right)+\beta \times Rate\left(term\right)\\ +\gamma \times SecScore\left(sec\right)+\delta \times SenScore\left(sen\right)\end{array}$$

Here, ‘term’ is the identified metadata entity, while ‘sec’ and ‘sen’ are, respectively, the section of the publication (e.g., Introduction, Materials and Methods, etc.) and the sentence in which the term is found. ‘Freq’ calculates the frequency of ‘term’ by simply counting the number of times the term appears within the publication. ‘Rate’ computes how often NeuroMorpho.Org uses the term by dividing the number of times a group of neural reconstructions is annotated with that specific entity by the count of all group of reconstructions annotated by any entity within that metadata category.

‘SecScore’ returns the importance of the section in which the term is identified, assigning, for example, greater weight to Materials and Methods or Results than to Introduction or Discussion (Table 2). Figure legends are assigned the SecScore value of the section they belong to (typically Results). If a term is found in multiple sections within the publication, the maximum SecScore value is utilized.

**Table 2:** List of different sections considered in publications along with their relative importance for the modeling. “Summary” is considered synonymous with Abstract and “Conclusions” with Discussion. “Others” include Acknowledgments and References as well as any additional section

Section	Title	Abstract	Keywords	Introduction	Methods	Results	Discussion	Others
Importance	1.0	1.0	1.0	0.5	1.0	1.0	0.6	0.4

‘SenScore’ calculates the relevance of the sentences containing the term. For this purpose, we trained the logistic regression classifier Scikit-learn [30], using default parameters, on 375 sample sentences randomly selected from neuroscience articles associated with NeuroMorpho.Org data, and manually labeled as 0 or 1 based on their informativeness. This classifier reads the embedded sentences from the last layer of NeuroBERT (Fig. 3) and uses a sigmoid function to produce a likelihood value based on its structure. For example, the label ‘Species = rat’ in the sentence "experiment was performed on 55 adult male Sprague–Dawley rats" will have a higher value (SenScore = 0.85) than in the sentence "previous in vitro studies of adult rat have shown that correlation depends on the level of excitation" (SenScore = 0.40). If a term is found in multiple sentences within the publication, the maximum SenScore value is utilized.

### Model training and parameter settings

The ranking values (Freq, Rate, SenScore, and SecScore) were min–max normalized to the [0, 1] range. Their coefficient values [α, β, γ, δ] were optimized using grid search in [0, 1] interval with 0.05 incremental steps as those maximizing annotation performance (Table 3). We used default values for most BERT hyperparameters [11], except for the following. The learning rate was set at 0.00002 upon testing the 5 recommended values and a batch size of 8 was used after comparing the results with a value of 16. We chose 10 as the number of training epochs, which produced the best results among all values from 1 to 50. Model training and optimization were performed using Python 3.10 under Linux operating system on a Tesla K80 GPU with 32 GB of RAM and lasted 3 days. The full list of packages and libraries used is available at https://gitlab.orc.gmu.edu/kbijari/neuroner-api.

**Table 3:** Best performing parameters for the model

Parameter	*α*	*β*	*γ*	*δ*
Value	0.20	0.25	0.35	0.20

## Results

We use the well-known information retrieval metric, accuracy [24], to quantify the metadata extraction and suggestion performance as well its robustness relative to the training dataset size. Moreover, we report the outcome of a labor analysis estimating the amount of manual curation that the automated suggestion system can save in the day-to-day curation of forthcoming NeuroMorpho.Org content. Finally, we introduce an API to allow integration of the metadata extraction and suggestion system with other software components as well as a graphical interface enabling user-friendly open access.

### Metadata extraction from scientific literature

**Fig. 4:**
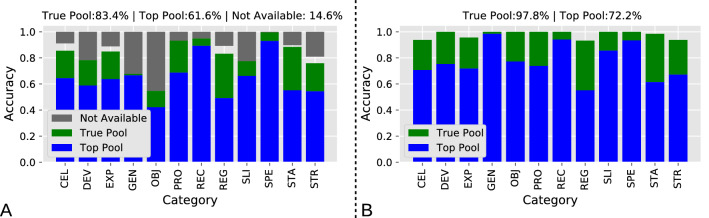
Accuracy of metadata extraction from neuroscience articles by metadata category (see Table 1 for abbreviations). A. The Top Pool (blue bars) consists of the highest ranking recommended terms. The True Pool (green bars) includes all terms in the suggestion list regardless of rank. Gray bars from the top indicate the proportion of target metadata terms for which the article provides no information. The numerical value reported above the bar plot are averages of all metadata categories. B. Same data as in panel A with Not available (gray bars) removed and other values scaled accordingly

We first tested the performance of the metadata extraction and suggestion system against 812 articles for which the entities of interest had been manually curated and accessible on NeuroMorpho.Org (Fig. 4). For each article and metadata category, we checked whether the set of terms suggested by the system included the manually annotated term (considered here as gold standard). If so, we further checked whether the target term was the highest ranking one within all suggested terms. In this analysis, we refer to True Pool as the cases in which the correct term was identified by the system but did not rank at the top; and to Top Pool as the cases in which the correct term was identified as the highest ranking of all identified terms. Thus, the True Pool percentage quantifies the *suggestion* performance for computer-assisted human annotation, while the Top Pool percentage quantifies the *recommendation* performance of a fully automated machine annotation. Over all metadata category, the system achieved a suggestion (computer-assisted) performance of ~ 84% and a recommendation (fully automated) performance of ~ 62% (Fig. 4A). These proportions varied widely by metadata category. The species, for instance, was correctly recommended ~ 95% of the times and always suggested in the remaining 5%. The objective type, in contrast, was correctly recommended just ~ 45% of the times and merely suggested only in another 10% of cases. When we investigated the missed labels, we discovered that in most instances the article simply did not report the relevant information (marked as “Not-Available” in Fig. 4A). In those cases, NeuroMorpho.Org either labeled the corresponding metadata categories as Not Reported or Not Available, or else obtained the correct label through personal correspondence with the authors. If excluding the labels not available in the publication text, the suggestion performance approaches 100% in most metadata categories and exceeds 90% in all of them (~ 98% overall), with a corresponding recommendation performance of ~ 72% (Fig. 4B).

**Fig. 5:**
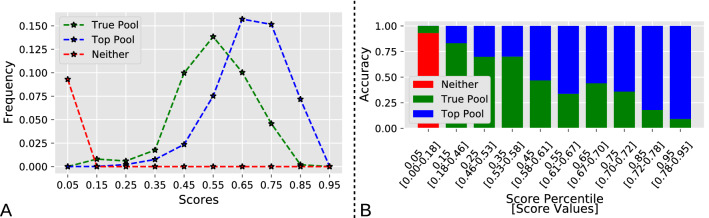
**A** Frequency of term scores for terms in the Top Pool, True Pool, and in neither (missed labels). **B** Accuracy as a function of term score and corresponding term score percentile

We then analyzed the distribution of scores utilized in the ranking and how they differed among the Top Pool, True Pool, and missed labels (Fig. 5). As expected, the Top Pool had scores mostly distributed toward high values, whereas the missed labels had the lowest value scores, with the True Pool characterized by intermediate values (Fig. 5A). The Top Pool and True Pool score distributions overlapped substantially at intermediate score values. However, the overall score distribution was not distributed uniformly over all [0, 1] values. Instead, the majority of scores fell in the [0.4, 0.9] range. Thus, we ranked all scores and analyzed the accuracy of terms by score percentile (Fig. 5B). This analysis indicated that the label predictions in the bottom 10% of scores, up to a score value of 0.18, are mostly incorrect and can thus be safely discarded. In contrast, label predictions in the top 10% of scores, above a score value of 0.78, are almost entirely in the Top Pool, and can thus be utilized for fully automated annotation. For labels in intermediate value percentiles, the balance of Top Pool vs. True pool shifts gradually. This means, for example, that approximately two-thirds of scores in the 50–90 percentiles can be trusted as correct labels.

**Fig. 6:**
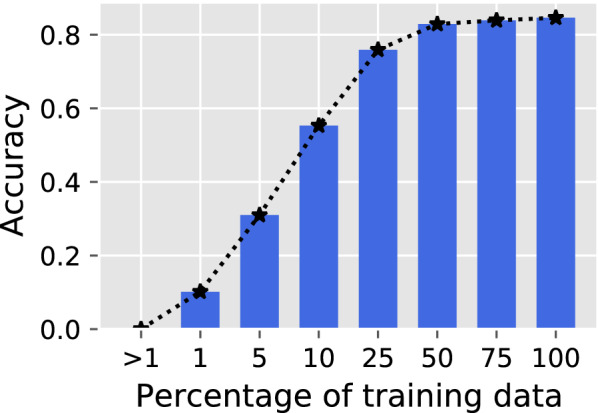
Overall accuracy as a function of training dataset sizes

Next, we investigated whether the performance of the metadata extraction and suggestion system was limited by the amount of training data. To this aim, we analyzed performance robustness while progressively reducing the training data size. Specifically, we used different percentages of the annotated sentences to train the metadata extraction algorithm and quantified in each case True Pool accuracy (Fig. 6).

While the performance, as expected, initially increased with the amount of training data, it did so steeply, effectively reaching a plateau at approximately 50% of the training dataset size used in this work. This means that greater amounts of training data would be unlikely to further improve the accuracy of metadata extraction using the same architecture.

### Labor automation analysis

**Fig. 7:**
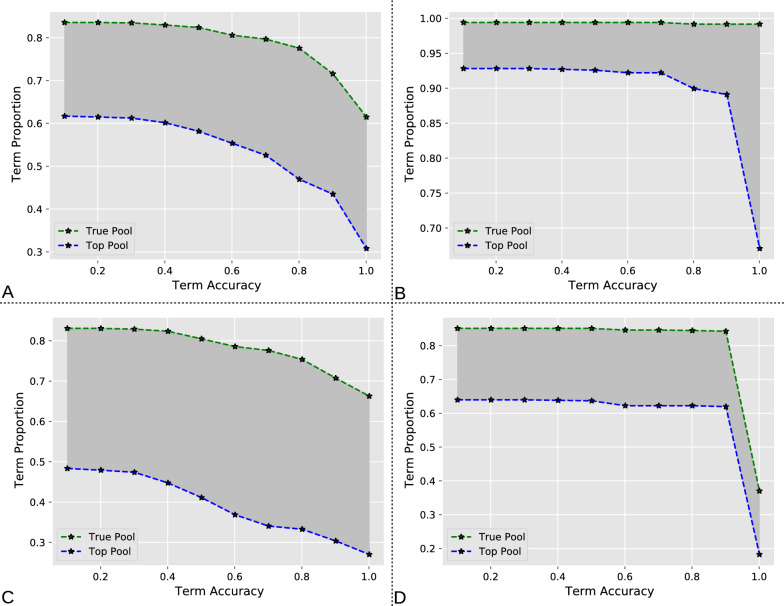
Labor automation analysis. **A** Fraction of labels identified by the metadata extraction system across all metadata categories as a function of identification accuracy. The green curves represent all suggested terms regardless of rank (True Pool), while the blue curves represented the recommended terms only if they appear at the highest rank (Top Pool). Same analysis broken down by species (**B**), brain region (**C**), and experimental condition (**D**)

An important practical consideration of a semi-automated labeling system is the trade-off between the proportion of data that it can extract and the resultant accuracy. To characterize this trade-off, we examined the accuracy of all individual terms extracted in the test data. The proportion of terms captured within a given accuracy is indicative of the amount of work that could be saved through automatic extraction. In particular, this analysis quantifies the potential labor saving of semi-automated suggestions and fully automated recommendations as a function of desired accuracy (Fig. 7).

If we require, for example, an overall accuracy of 75% or more, full automation could identify the exact target entity for 50% of terms, whereas a hybrid computer-assisted suggestion system would include the right term within a pool of suggestions in 80% of cases, leaving only the remaining 20% for human annotators to find from scratch (Fig. 7A). Notably, labor saving differs by metadata dimension. For instance, with an accuracy of 90%, the extraction system could pinpoint 90% of the target species (Fig. 7B) as the first choice; however, it could only find 60% of the experimental conditions (Fig. 7D). This means that the metadata suggestions could be accepted with different levels of confidence depending on their category.

### Integration with other portals and deployment for NeuroMorpho.Org

**Fig. 8:**
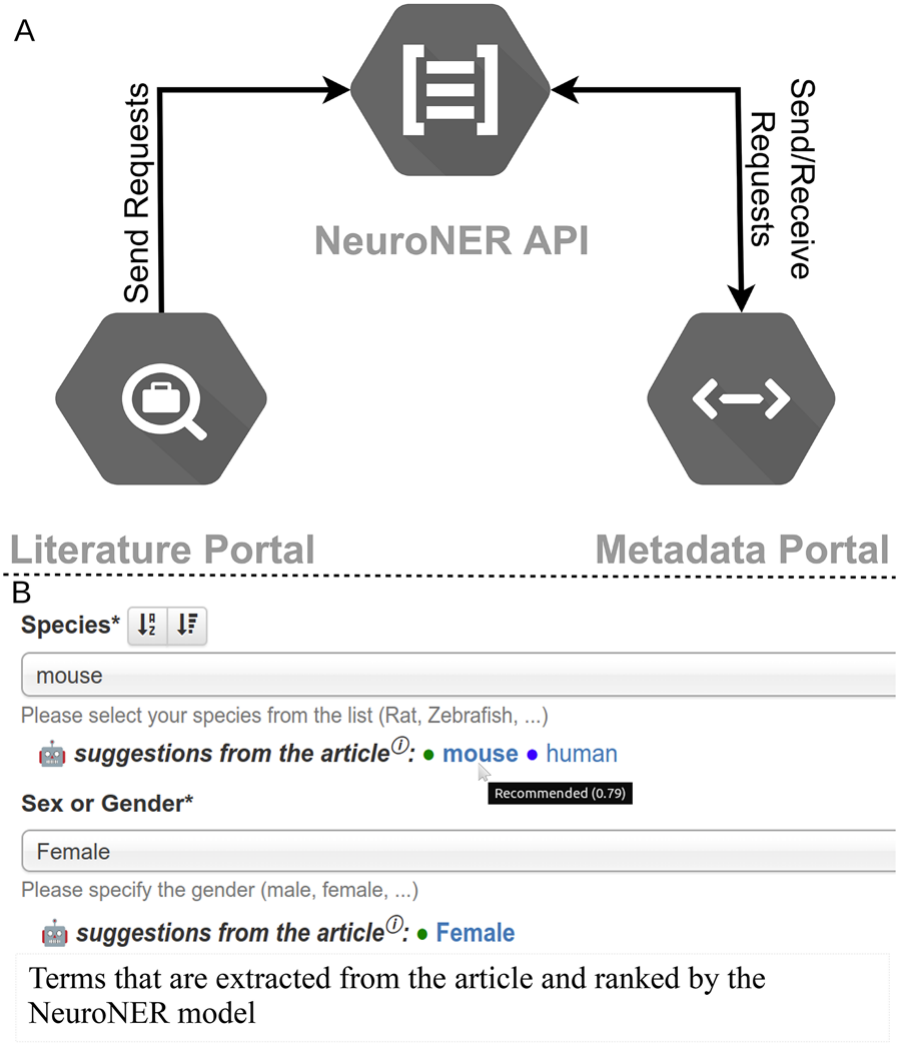
**A** API interactions between NeuroMorpho.Org functionalities. When a relevant article is identified, the literature portal sends a request to the NeuroNER API with a pointer to the article identifier. The API then processes the request and sends the extraction labels back to the metadata portal. **B** Enhanced graphical user interface of the metadata portal. For different metadata dimensions (e.g., species and sex), the web form now shows a sorted list of clickable suggestions automatically extracted from the article text. The color coding reflects the level of confidence associated with the suggestion: green (recommended): score ≥ 0.78; blue (suggested): 0.78 > score ≥ 0.45; scores below 0.45 are discarded

One main objective of this metadata extraction system is to interact with other previously developed NeuroMorpho.Org functionalities, specifically the literature portal [25] and the metadata management systems [7]. Accordingly, we designed an API, named NeuroNER, that hosts the trained architecture and awaits requests from other servers to fulfill (Fig. 8). In particular, when the literature portal finds a new relevant publication for NeuroMorpho.Org, it sends to NeuroNER links to the full-text and related information, including PubMed ID, digital object identifier, publisher, authors, and affiliations. After receiving the request, the API then processes these data to extract the metadata labels from the full text, sort them based on their score, and saves the resultant information in a local database. When a user requests this information on the metadata portal, NeuroNER posts back the JSON formatted data (Fig. 8A).

We have also upgraded the metadata portal to display the information received from NeuroNER as a list of suggestions for different metadata categories, sorted and color-coded by score (Fig. 8B). Hovering over the suggestions with the mouse cursors pops up the score value and color codes. Clicking on a suggestion selects that label and overwrites previous annotations in the same category.

## Discussion

Proper curation, interpretation, and analysis of neuroscience information are crucial for the continuous growth of neuroinformatics efforts, such as advanced data repositories and large-scale brain modeling projects. However, manually sifting through thousands of peer-reviewed articles for detailed metadata is a burden with small or no reward. To facilitate this process for the practitioners, we adapted and expanded a state-of-the-art deep learning tool to implement a text mining application for detecting, tagging, and ranking neuroscience named entities in the context of NeuroMorpho.Org. Our work demonstrates that increasingly widespread machine learning techniques in natural language processing are useful for extracting neuroscience information from the unstructured text of research publications. Progressively automating metadata retrieval can considerably aid the efforts of neuroscientists in their daily curation tasks. This work characterized the application of information extraction for the specific curation needs of NeuroMorpho.Org. However, most other knowledge data repositories and informatics projects that rely on literature-mining and information extraction can likewise benefit from a similar approach and implementation as described here. A prime example in this regard is the curated knowledge base of neuron types in the hippocampus circuits, Hippocampome.Org [45].

Several relevant efforts have been described to aid the metadata extraction and annotation process. Those previous works differ from the system described in this report in terms of design, implementation, resource management or usage, and do not fully satisfy the curation needs of NeuroMorpho.Org. For example, PubTator is a web-based application that assists in the prioritization, curation, and annotation of articles with a focus on molecular concepts, such as genes, proteins, chemicals, and mutations [43]. Its usage of predefined packages, dictionaries, and rules to extract bio-entity terms and their relations makes it impractical to extend to a different domain. Another project, WhiteText, uses NLP to recognize solely mentions of brain anatomy in neuroscience text with the goal of automatically extracting regional connectivity information, without covering other metadata categories [13]. The widely used ModelDB repository of neuroscience models [17] implemented an automated suggestion system using manually curated regular expression-based rules to facilitate annotation [26]. This approach yielded 79% precision when tagging metadata from abstracts, but only 41% from the full text, which is insufficient for the needs of NeuroMorpho.Org. The odMLtables is a complement to the open metadata Markup Language (odML) framework for managing neurophysiological metadata [41]. This effort focuses on unifying the format utilized to annotate metadata rather than with information extraction per se.

Unrestricted access to mined metadata on publicly shared repositories is vital to enable reproducibility, replicability, further scientific exploration, and data-driven computational modeling [5, 14, 33]. Within the domain of neural morphology, recent developments include detailed statistical analyses enabled by machine learning [8] and tools for organizing large amounts of data based on arbitrary combinations of user-selected metadata [1]. More broadly, the prominence of such endeavors is continuously growing in neuroscience [3].

In the longer term, we envision a fully autonomous system capable of automatically extracting all relevant metadata for a dataset from the related peer-reviewed article without any human input and with the accuracy of domain experts. The work described in this report represents substantial progress toward this goal and reveals the extent of the remaining challenges. On the one hand, our existing system demonstrated excellent performance in identifying the correct label when it is mentioned in the publication full text (~ 98% accuracy). On the other, the system is not yet equipped to recognize when the article does not provide suitable information to annotate a given metadata category (not reported or not applicable cases), which occurs for 14.6% of entries. Moreover, in a majority, but not in the totality, of cases does the target label rank at the top of the scores among the identified term for that metadata category. As a combined effect of these factors, at present the overall accuracy for fully automated usage falls to ~ 62%, which is insufficient for the expectations of the NeuroMorpho.Org community. Despite this shortcoming, the described system is already useful as a computer-assisted suggestion system, and when deployed as such can halve the annotation labor.

Our analysis demonstrated that the performance limits of this approach cannot be overcome by simply increasing the training dataset size. Thus, we are considering alternative strategies to extend this effort in future upgrades. One long-term possibility is to incorporate NeuroMorpho.Org’s new similarity search engine [22] to augment the metadata suggestions based on the resemblance of the actual neural reconstructions. The rationale behind this idea is that neurons with similar morphological attributes would tend to have matching metadata characteristics, e.g., in terms of animal species, brain region, and cell type, but also experimental protocol [38]. An alternative or additional improvement could leverage statistical correlations within and among different metadata dimensions, which can be extracted from the NeuroMorpho.Org database [32]. These potentially predictive relations could reflect hard biological constraints (e.g., if the neuron type is pyramidal, the anatomical region cannot be retina) or soft sub-community preferences (e.g., Knossos is the most popular reconstruction software to skeletonize neurons from electron microscopy).

The semi-automated suggestion system introduced in this work constitutes a foundational first step in the direction of seamless, machine-driven metadata annotation for NeuroMorpho.Org. Increasingly autonomous curation reduces the burden for human experts and enables continuous growth toward ever larger datasets, ushering in the big science era of computational neuroscience.

## Data Availability

Project name: NeuroMorpho.Org Semi-Automatic Metadata Extraction and Annotation. Project home page: http://cng-nmo-meta.orc.gmu.edu/ and http://cng-nmo-dev6.orc.gmu.edu/. Operating system: Platform independent. Programming languages: Python, HTML, Javascript. Other requirements: Python 3, Flask 2.0, Nginx. License: GPL 3.0. Datasets and source code: https://gitlab.orc.gmu.edu/kbijari/neuroner-api.

## Abbreviations

API: Application Programming Interface
NER: Named Entity Recognition
NLP: Natural Language Processing
BERT: Bidirectional Encoder Representations from Transformers
JSON: Java Script Object Notation
